# A769662 Inhibits Insulin-Stimulated Akt Activation in Human Macrovascular Endothelial Cells Independent of AMP-Activated Protein Kinase

**DOI:** 10.3390/ijms19123886

**Published:** 2018-12-05

**Authors:** Anastasiya Strembitska, Sarah J. Mancini, Jonathan M. Gamwell, Timothy M. Palmer, George S. Baillie, Ian P. Salt

**Affiliations:** 1 Institute of Cardiovascular and Medical Sciences, College of Medical, Veterinary and Life Sciences, University of Glasgow, Glasgow G12 8QQ, UK; a.strembitska.1@research.gla.ac.uk (A.S.); sarah.mancini@glasgow.ac.uk (S.J.M.); jonathan.gamwell@rdm.ox.ac.uk (J.M.G.); tim.palmer@hyms.ac.uk (T.M.P.); george.baillie@glasgow.ac.uk (G.S.B.); 2 Radcliffe Department of Medicine, University of Oxford, Oxford OX3 9DU, UK; 3 Centre for Atherothrombosis and Metabolic Disease, Hull York Medical School, University of Hull, Hull HU6 7RX, UK

**Keywords:** AMP-activated protein kinase, protein kinase B, Akt, insulin signalling, A769662, endothelial function

## Abstract

Protein kinase B (Akt) is a key enzyme in the insulin signalling cascade, required for insulin-stimulated NO production in endothelial cells (ECs). Previous studies have suggested that AMP-activated protein kinase (AMPK) activation stimulates NO synthesis and enhances insulin-stimulated Akt activation, yet these studies have largely used indirect activators of AMPK. The effects of the allosteric AMPK activator A769662 on insulin signalling and endothelial function was therefore examined in cultured human macrovascular ECs. Surprisingly, A769662 inhibited insulin-stimulated NO synthesis and Akt phosphorylation in human ECs from umbilical veins (HUVECs) and aorta (HAECs). In contrast, the AMPK activators compound **991** and AICAR had no substantial inhibitory effect on insulin-stimulated Akt phosphorylation in ECs. Inhibition of AMPK with SBI-0206965 had no effect on the inhibition of insulin-stimulated Akt phosphorylation by A769662, suggesting the inhibitory action of A769662 is AMPK-independent. A769662 decreased IGF1-stimulated Akt phosphorylation yet had no effect on VEGF-stimulated Akt signalling in HUVECs, suggesting that A769662 attenuates early insulin/IGF1 signalling. The effects of A769662 on insulin-stimulated Akt phosphorylation were specific to human ECs, as no effect was observed in the human cancer cell lines HepG2 or HeLa, as well as in mouse embryonic fibroblasts (MEFs). A769662 inhibited insulin-stimulated Erk1/2 phosphorylation in HAECs and MEFs, an effect that was independent of AMPK in MEFs. Therefore, despite being a potent AMPK activator, A769662 has effects unlikely to be mediated by AMPK in human macrovascular ECs that reduce insulin sensitivity and eNOS activation.

## 1. Introduction

Endothelial cells (ECs) are essential for modulation of vascular homeostasis and signal transduction [1], including the production and regulation of vascular tone, modulation of inflammatory responses and maintenance of an anti-atherogenic phenotype of vascular smooth muscle cells (VSMCs) [1]. Being the downstream target of phosphoinositide (PI) 3-kinase (PI3K), protein kinase B (Akt) is one of the key kinases regulating cell survival, cell-cycle progression and metabolism [2]. Under physiological conditions, insulin [3], VEGF [4] or IGF1 [5] stimulation rapidly leads to increased Akt activity by increasing Ser473 and Thr308 phosphorylation.

In healthy subjects, insulin acts as a vasodilator, stimulating calcium-independent NO synthesis in cultured human aortic ECs (HAECs) through Akt-mediated phosphorylation of endothelial nitric oxide synthase (eNOS) Ser1177 and Ser615 [6]. However, in murine models of diabetes and people with diabetes, impaired insulin-stimulated blood flow and NO bioavailability have been demonstrated [7]. Decreased NO bioavailability leads to endothelial dysfunction in animal models and human subjects, increasing risk of cardiovascular events and pro-inflammatory signalling in the vasculature [8].

AMP-activated protein kinase (AMPK) is a heterotrimeric serine/threonine protein kinase sensitive to intracellular AMP levels which acts as a master regulator of energy metabolism [9,10]. AMPK is activated in response to an increase in the intracellular (AMP + ADP):ATP ratio and simultaneous AMPKα Thr172 phosphorylation by the upstream kinases liver kinase B1 or calcium/calmodulin-dependent protein kinase kinase β [9,10]. Activated AMPK inhibits anabolic processes which consume ATP [9,10], reduces inflammation [11,12], inhibits endothelial cell proliferation [13], stimulates mitochondrial biogenesis [14], and increases insulin sensitivity [9,15], making it an attractive pharmacological target for diabetes and cardiovascular pathologies [10]. There are 12 differentially expressed AMPK isoforms, each composed of catalytic α1/α2, and regulatory β1/β2 and γ1/γ2/γ3 subunits [9,10], allowing a specialised cellular and systemic response to different metabolic stimuli [10].

Previous studies have reported that AMPK activation improved insulin sensitivity and energy homeostasis, and attenuated inflammatory signalling in insulin-sensitive tissues, such as muscle [14], liver [16,17] and adipose tissue [11,18] as well as ECs [12,15]. Several AMPK activators have been shown to increase NO synthesis in HAECs in an AMPK-dependent manner [10]. Nevertheless, it is unclear whether all AMPK activators improve insulin sensitivity and vascular function, as previous studies have largely been conducted using a variety of compounds that activate AMPK by altering cellular nucleotide ratios, including rosiglitazone, resveratrol, metformin and canagliflozin or mimic AMP, such as AICAR (5-amino-4-imidazolecarboxamide ribonucleoside) [12,19,20,21,22].

In the last 15 years, there has been considerable effort to develop AMPK-selective small-molecule activators, resulting in the development of A769662 [23], the discovery of salicylate as an AMPK activator and, more recently, the development of compound **991**—a compound 5–10-fold more potent than A769662 [24]. A769662 is an allosteric activator that binds complexes containing AMPKβ1, also inhibiting AMPKα Thr172 dephosphorylation [24,25]. Since being first described as an AMPK activator, some AMPK-independent effects of A769662 have been reported [26,27,28,29], yet it remains a commonly utilised pharmacological tool for selective AMPK activation. The effects of A769662 on insulin signalling and insulin-stimulated Akt/eNOS axis activation in primary human ECs were therefore determined.

## 2. Results

### 2.1. Insulin-Stimulated Signalling and NO Production are Reduced by A769662 in Human Endothelial Cells

**Figure 1:**
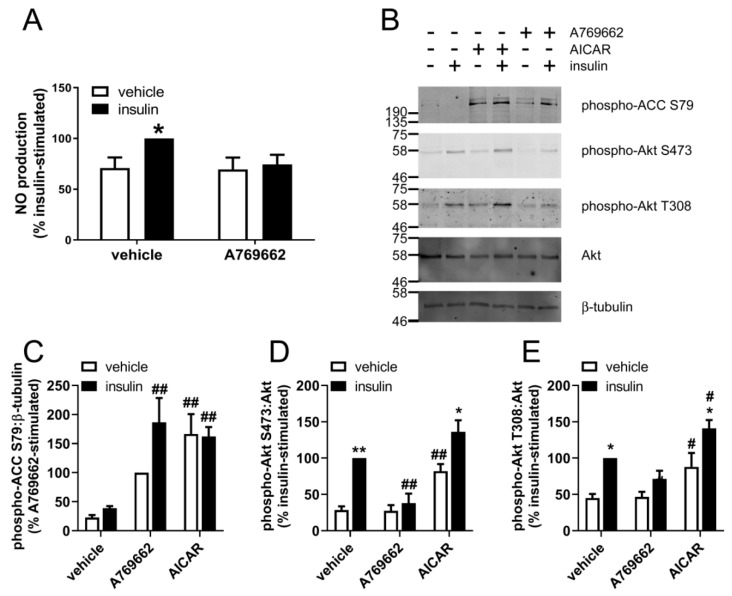
The effect of A769662 on insulin-stimulated NO production and Akt phosphorylation in HUVECs. (**A**) HUVECs were stimulated with A769662 (50 µM, 45 min) prior to insulin (1 µM, 15 min), conditioned media collected and NO production assessed. Data (mean ± SEM) shown from three independent replicates. (**B**–**E**) HUVECs were stimulated with A769662 (50 µM, 45 min) or AICAR (2 mM, 45 min) prior to insulin (51 µM, 15 min) and cell lysates prepared. Proteins were resolved by SDS-PAGE and immunoblotted with the antibodies indicated. (**B**) Representative immunoblots from three biological replicates with molecular weight markers indicated. Phospho-Akt Thr308 protein levels were assessed by stripping and re-probing the membranes. Densitometric quantification of (**C**) ACC, (**D**) Akt Ser473 and (**E**) Akt Thr308 phosphorylation normalised to β-tubulin or Akt (mean ± SEM). * *p* < 0.05, ** *p* < 0.01, relative to absence of insulin. # *p* < 0.05, ## *p* <0.01 relative to absence of AMPK activator.

Stimulation of HUVECs with concentrations of insulin above 0.1 µM robustly stimulated phosphorylation of Akt at Ser473 and Thr308 (Supplementary Figure S1), similar to concentrations of insulin previously demonstrated to be required for insulin-stimulated NO synthesis in cultured endothelial cells [30]. All subsequent experiments were therefore conducted using 1 µM insulin. To examine the effect of A769662 on HUVEC insulin signalling, cells were preincubated in the presence or absence of A769662 prior to stimulation with insulin and NO synthesis and the extent of Akt phosphorylation assessed. Insulin modestly increased NO synthesis, yet insulin-stimulated NO synthesis was lost upon preincubation with A769662 (Figure 1A).

**Figure 2:**
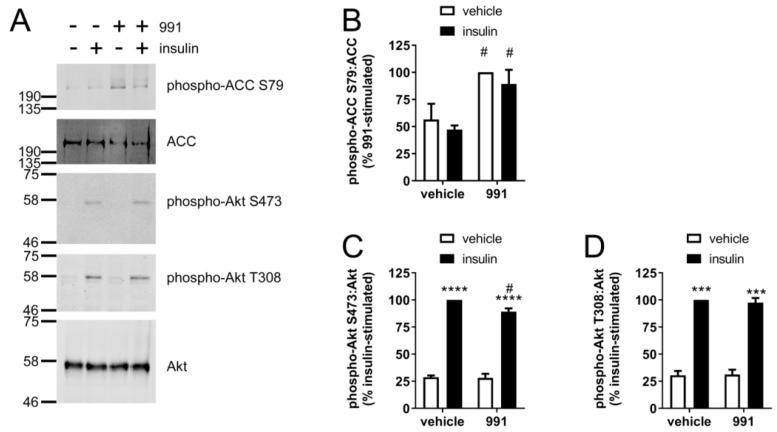
The effect of compound **991** on insulin-stimulated Akt phosphorylation in HUVECs. HUVECs were stimulated with compound **991** (5 µM, 45 min) prior to insulin (1 µM, 15 min) and cell lysates prepared. Proteins were resolved by SDS-PAGE and immunoblotted with the antibodies indicated. (**A**) Representative immunoblots from three biological replicates with molecular weight markers indicated. Phospho-Akt Thr308 protein levels were assessed by stripping and re-probing the membranes. Densitometric quantification of (**B**) ACC Ser79, (**C**) Akt Ser473 and (**D**) Akt Thr308 phosphorylation normalised to ACC or Akt (mean ± SEM). *** *p* < 0.001, **** *p* <0.0001 relative to absence of insulin. # *p* <0.05 relative to absence of compound **991**.

A769662 (50 μM, 45 min) increased AMPK activity, assessed by immunoblotting of AMPK-specific ACC (acetyl CoA carboxylase) Ser79 phosphorylation (Figure 1B,C), yet insulin had no effect on ACC phosphorylation. The inhibition of NO production was associated with markedly reduced insulin-stimulated phosphorylation of Akt at Ser473 and Thr308 (Figure 1D,E). In contrast, the AMPK activator AICAR, which is converted to the AMP mimetic ZMP in cells, increased basal and insulin-stimulated Akt Ser473 and Thr308 phosphorylation (Figure 1D,E), despite activating AMPK to a similar degree as assessed by ACC Ser79 phosphorylation (Figure 1C). Furthermore, the direct AMPK activator compound **991**, which allosterically activates AMPK at the same site as A769662 [31], had no effect on basal or insulin-stimulated Akt Thr308 phosphorylation, and only modestly reduced insulin-stimulated Akt Ser473 phosphorylation by 10% despite activating AMPK to a similar extent (Figure 2).

**Figure 3:**
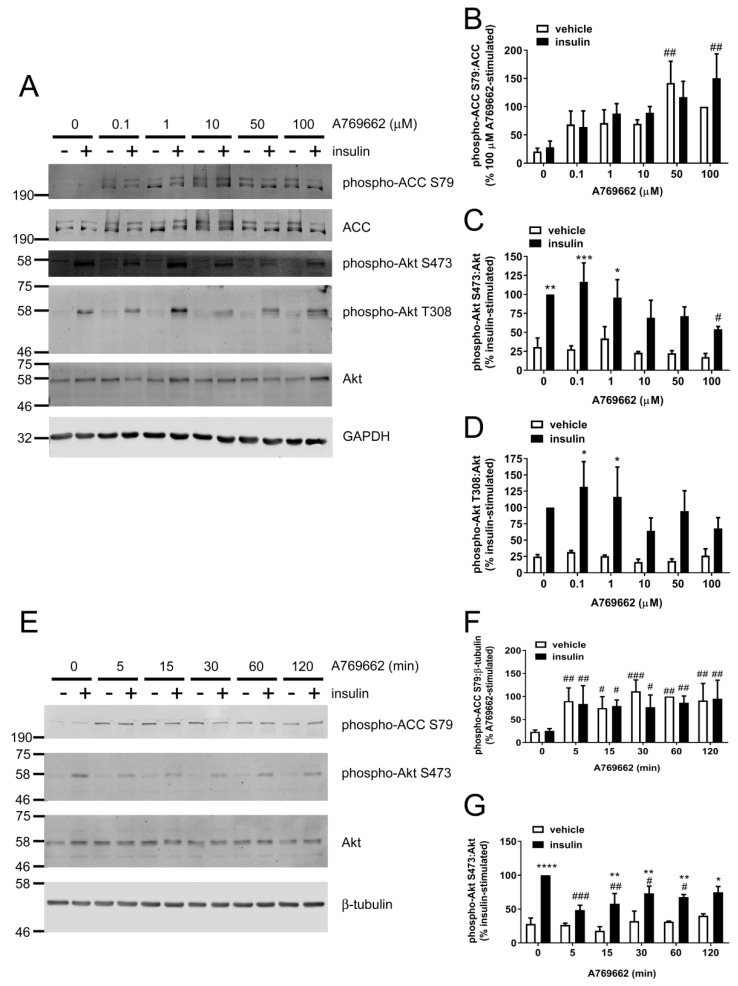
A769662 decreases Akt phosphorylation in a concentration- and time-dependent manner in HUVECs. HUVECs were stimulated with (**A**–**D**) the indicated concentrations of A769662 for 45 min or (**E**–**G**) 50 µM A769662 for the indicated durations prior to insulin (1 µM, 15 min). Cell lysates were prepared and immunoblotted with the antibodies indicated. (**A**,**E**) Representative immunoblots from three independent biological replicates with molecular weight markers indicated. Total ACC protein level was assessed by stripping and re-probing the membranes. Densitometric quantification of (**B**,**F**) ACC Ser79, (**C**,**G**) Akt Ser473 and (**D**) Akt Thr308 phosphorylation (mean ± SEM). * *p* < 0.05, ** *p* < 0.01, *** *p* < 0.001, **** *p* < 0.0001 relative to absence of insulin. # *p* < 0.05, ## *p* < 0.01, ### *p* < 0.001 relative to absence of A769662.

To examine the relationship between AMPK activation and inhibition of insulin-stimulated Akt phosphorylation by A769662, the concentration dependence of either effect of A769662 was assessed. Significant A769662-mediated stimulation of ACC phosphorylation was achieved with 50–100 μM A769662 (Figure 3A,B). A769662 decreased both insulin-stimulated Akt Ser473 and Thr308 phosphorylation in HUVECs in a concentration-dependent manner (Figure 3A), whereby 100 µM A769662 significantly inhibited Akt S473 phosphorylation and the statistical significance of insulin-stimulated Akt phosphorylation at either site was lost at concentrations above 10 µM A769662 (Figure 3C,D).

Furthermore, the time dependence of the inhibitory effect of A769662 on insulin-stimulated Akt phosphorylation was assessed. A769662 (50 µM) rapidly stimulated ACC phosphorylation in HUVECs within 5 min, an effect that was sustained for at least 2 h (Figure 3E,F). The inhibitory effect of A769662 on insulin-stimulated Akt Ser473 phosphorylation occurred similarly rapidly within 5 min and was sustained for 1 h (Figure 3E,G).

**Figure 4:**
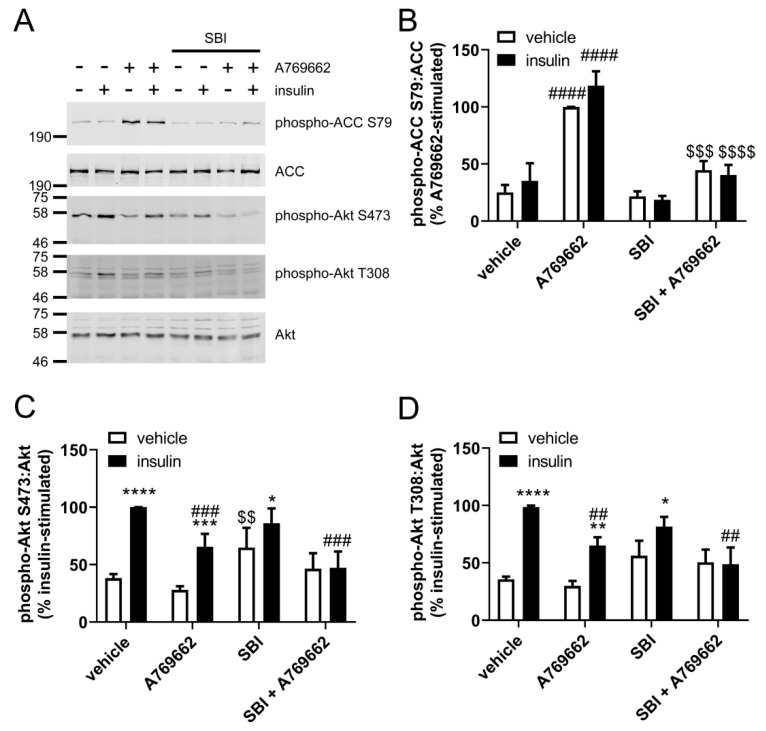
SBI-0206965 inhibits A769662-stimulated AMPK activation without altering inhibition of insulin-stimulated Akt phosphorylation in HUVECs. HUVECs were stimulated with A769662 (50 µM, 45 min) prior to insulin (1 µM, 15 min) after preincubation in the presence or absence of SBI-0206965 (SBI, 30 µM, 30 min) and cell lysates prepared. Proteins were resolved by SDS-PAGE and immunoblotted with the antibodies indicated. (**A**) Representative immunoblots from six biological replicates with molecular weight markers indicated. Densitometric quantification of (**B**) ACC, (**C**) Akt Ser473 and (**D**) Akt Thr308 phosphorylation normalised to total ACC or Akt (mean ± SEM). * *p* < 0.05, ** *p* < 0.01, *** *p* < 0.001, **** *p* < 0.0001 relative to absence of insulin. ## *p* < 0.01, ### *p* < 0.001, #### relative to absence of A769662. $$ *p* < 0.01, $$$ *p* < 0.001, $$$$ *p* < 0.0001 relative to absence of SBI-0206965.

To determine whether the inhibition of insulin-stimulated Akt phosphorylation in ECs was AMPK-dependent, similar experiments were conducted after prior incubation in the SBI-0206965, which has recently been described as a selective inhibitor of AMPK [32]. Preincubation with 30 µM SBI-0206965 completely inhibited A769662-stimulated ACC Ser79 phosphorylation (Figure 4A,B), yet had no effect on the inhibition of insulin-stimulated Akt phosphorylation at Ser473 or Thr308 (Figure 4A,C,D), further indicating that the inhibitory effect of A769662 was AMPK-independent.

### 2.2. Insulin Signalling and Insulin-Stimulated NO Production are Significantly Decreased in A769662-Treated HAECs

**Figure 5:**
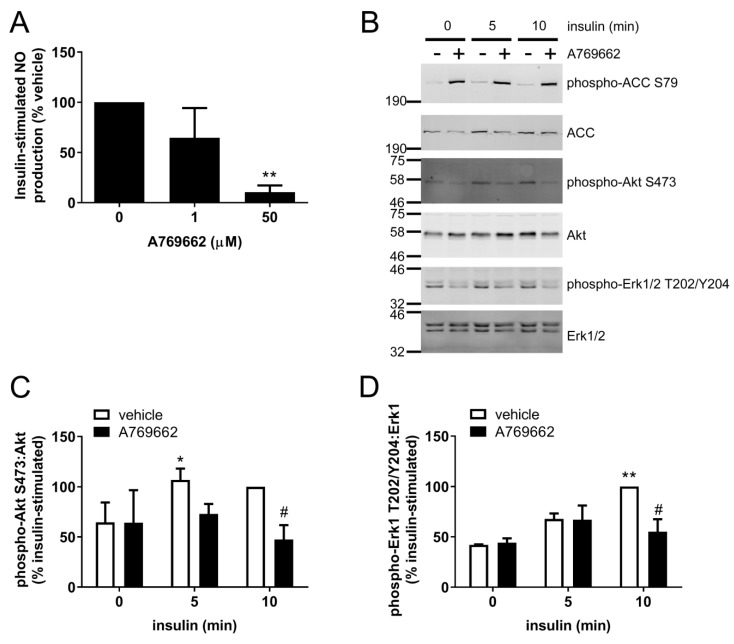
The effect of A769662 on insulin-stimulated NO synthesis and signalling in HAECs. (**A**) HAECs were stimulated with the indicated concentrations of A769662 prior to insulin stimulation (1 µM, 15 min). Conditioned media was collected and NO production assessed. (**B**–**D**) HAECs were incubated with 50 µM A769662 for 45 min prior to insulin stimulation (1 µM) for the indicated durations. Cell lysates were prepared and immunoblotted with the antibodies indicated. (**B**) Representative immunoblots from three independent biological replicates with molecular weight markers indicated. (**C**,**D**) Densitometric quantification (mean ± SEM) of (**C**) Akt Ser473 or (**D**) Erk1 Thr202/Tyr204 phosphorylation normalised to total Akt or Erk1 levels respectively (mean ± SEM). * *p* < 0.05, ** *p* < 0.01 relative to absence of insulin. # *p* < 0.05, relative to absence of A769662.

To determine whether this inhibition of insulin signalling was conserved in ECs from other regions of the vasculature, insulin-stimulated NO synthesis and signalling were assessed in HAECs. A769662 (50 μM) markedly inhibited insulin-stimulated NO synthesis (Figure 5A), an effect associated with reduced insulin-stimulated phosphorylation of Akt Ser473 (Figure 5B,C). To examine whether insulin signalling through an alternative pathway independent of PI3K was influenced by A769662, insulin-stimulated Erk1/2 phosphorylation was assessed. Intriguingly, A769662 significantly inhibited insulin-stimulated Erk1/2 phosphorylation (Figure 5B,D).

To examine whether the inhibitory action of A769662 on insulin-stimulated Akt phosphorylation was observed in non-endothelial cell lines, similar experiments were conducted in the HeLa tumour and HepG2 hepatoma cell lines. Preincubation with A769662 (50 μM, 45 min) or compound **991** (5 µM, 60 min) significantly increased ACC Ser79 phosphorylation in both HeLa (Supplementary Figure S2) and HepG2 cells (Supplementary Figure S3) to a similar extent. Unlike HUVECs, preincubation with A769662 had no statistically significant effect on basal or insulin-stimulated Akt Ser473 phosphorylation in either cell line, although the statistical significance of the effect of insulin was lost upon preincubation with A769662 in HeLa cells (Supplementary Figure S2C,D). Compound **991** did not affect insulin-stimulated Akt Ser473 and Thr308 phosphorylation in HepG2 cells (Supplementary Figure S3C,D) yet intriguingly did increase insulin-stimulated Akt Ser473 and Thr308 phosphorylation in HeLa cells (Supplementary Figure S2C,D). The inhibition of insulin-stimulated Akt Ser473 phosphorylation by preincubation with A769662 therefore seems to be restricted to ECs, and it is not observed in insulin-sensitive human cell lines.

### 2.3. A769662 Inhibits Insulin-Stimulated Erk1/2 Phosphorylation in an AMPK-Independent Manner

**Figure 6:**
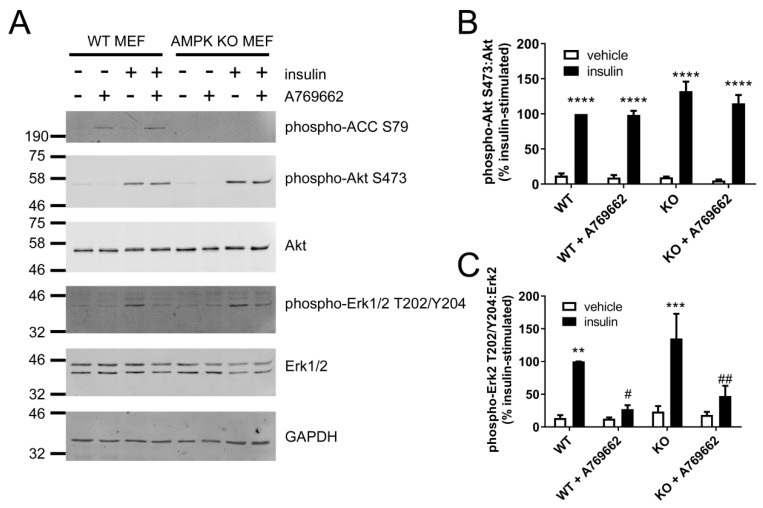
The effect of A769662 on insulin-stimulated Akt and Erk1/2 phosphorylation in wild-type and AMPK knockout MEFs. Wild-type (WT) and AMPK knockout (KO) MEFs were pre-incubated with A769662 (100 µM, 30 min) prior to insulin stimulation (1 µM, 15 min). Cell lysates were prepared, proteins resolved by SDS-PAGE and immunoblotted with the antibodies indicated. (**A**) Representative immunoblots from four independent biological replicates with molecular weight markers shown. (**B**,**C**) Densitometric quantification of (**B**) Akt Ser473 or (**C**) Erk2 Thr202/Tyr204 phosphorylation normalised to total Akt or Erk2 levels respectively (mean ± SEM). ** *p* < 0.01, *** *p* < 0.001, **** *p* < 0.0001 relative to absence of insulin. # *p* < 0.05, ## *p* < 0.01 relative to absence of A769662.

To further assess the AMPK-dependence of the inhibition of insulin-stimulated Akt and Erk1/2 phosphorylation by A769662, SV40-immortalised wild-type (WT) or AMPK knockout (KO) mouse embryonic fibroblasts (MEFs) [33] were stimulated with insulin after prior incubation in the presence or absence of A769662 (Figure 6). A769662 (100 µM, 30 min) robustly stimulated ACC phosphorylation in WT MEFs and ACC phosphorylation was undetectable in KO MEFs (Figure 6A). Insulin stimulated Akt Ser473 and Erk1/2 phosphorylation to a similar extent in cells from either genotype, yet A769662 had no effect on insulin-stimulated Akt Ser473 phosphorylation in either genotype (Figure 6B). In contrast, insulin-stimulated Erk1/2 phosphorylation was significantly inhibited by preincubation with A769662 in cells of either genotype (Figure 6C), indicating an AMPK-independent effect.

### 2.4. A769662 Inhibits IGF1-Stimulated Akt Ser473 Phosphorylation but Has No Effect on VEGF Signalling

**Figure 7:**
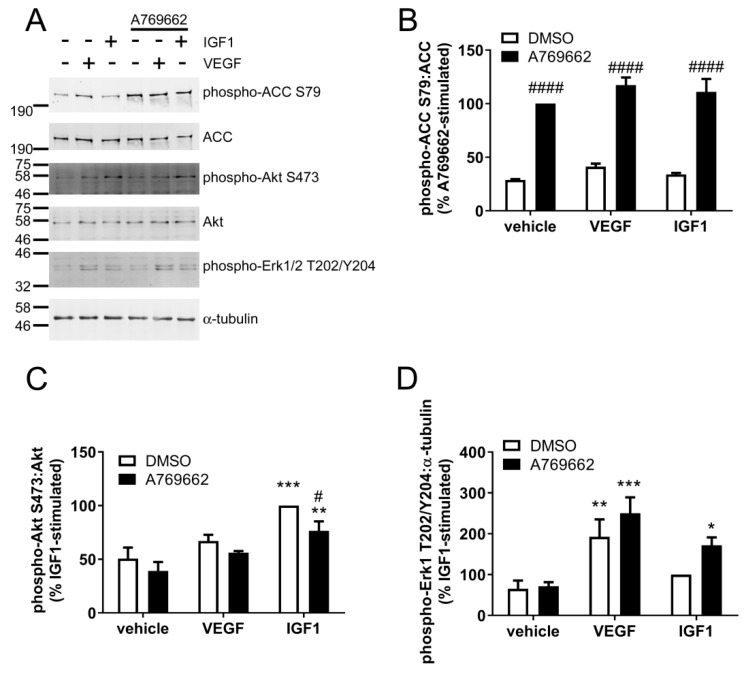
A769662 tends to attenuate IGF1-stimulated Ser473 Akt phosphorylation but has no effect on VEGF signalling in HUVECs. HUVECs were stimulated with A769662 (50 µM 45 min) prior to VEGF (10 ng/mL, 10 min) or IGF1 (25 ng/mL, 10 min). Cell lysates were prepared, proteins resolved by SDS-PAGE and immunoblotted with the antibodies indicated. (**A**) Representative immunoblots from four independent biological replicates with molecular weight markers indicated. Total ACC protein level was assessed by stripping and re-probing the membranes. (**B**–**D**) Densitometric quantification of (**B**) ACC Ser79, (**C**) Akt Ser473 or (**D**) Erk1 Thr202/Tyr204 phosphorylation normalised to total ACC, Akt or α-tubulin levels respectively (mean ± SEM). * *p* < 0.05, ** *p* < 0.01, *** *p* < 0.001 relative to absence of IGF1/VEGF. # *p* < 0.05, #### *p* < 0.0001 relative to absence of A769662.

To examine whether A769662 inhibits Akt phosphorylation in response to growth factors other than insulin in ECs, the effect of A769662 on IGF-1 and VEGF were assessed in HUVECs. VEGF (10 ng/mL, 10 min) stimulated a significant increase in Erk1 Thr202/Tyr204 phosphorylation, yet there was only a trend towards an increase in Akt Ser473 phosphorylation (Figure 7). In contrast, IGF1 (25 ng/mL, 10 min) significantly stimulated Akt Ser473 phosphorylation and tended to increase Erk1 Thr202/Tyr204 phosphorylation (Figure 7). Preincubation with A769662 significantly increased ACC Ser79 phosphorylation (Figure 7B) and modestly inhibited IGF1-stimulated Akt phosphorylation (Figure 7C). Although previous studies from our laboratory have shown VEGF stimulated AMPK activity after 5 min [34,35], stimulation with VEGF for 10 min only tended to increase ACC Ser79 phosphorylation in HUVECs (Figure 7B). This disparity may reflect the difference in incubation time, as in previous studies, VEGF stimulated transient ACC Ser79 phosphorylation that reached a maximum at 5 min and decreased rapidly after this point [34]. Furthermore, in contrast to the inhibitory action of A769662 on insulin-stimulated Erk1/2 phosphorylation observed in HAECs (Figure 5), IGF-1 significantly stimulated Erk1/2 phosphorylation only in the presence of A769662 (Figure 7D).

### 2.5. AMPK Complexes Containing α1 and β1 Isoforms Contribute the Majority of Total Cellular AMPK Activity in HAECs

**Figure 8:**
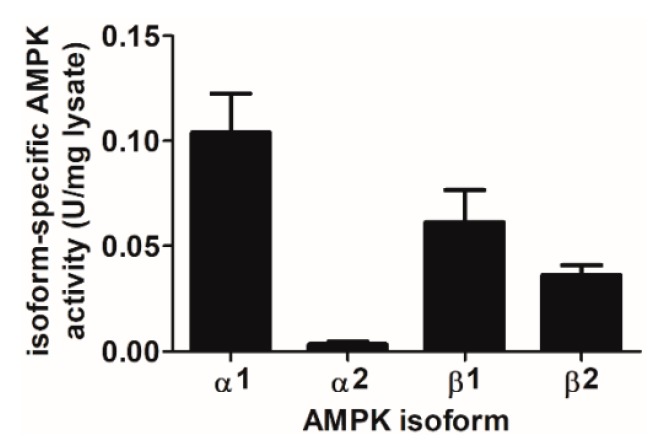
Activities of complexes containing specific AMPK isoforms in HAECs. AMPK was immunoprecipitated from HAEC lysates (100 µg) and AMPK activity assessed in immunoprecipitates by incorporation of ^32^P from [γ-^32^P]ATP into SAMS peptide. AMPK activity is presented as U/mg lysate protein (1 U = 1 nmol ^32^P incorporated into SAMS peptide/min). Results shown are mean ± SEM activity from three independent experiments.

A769662 and compound **991** selectively activate AMPK complexes containing the β1 regulatory subunit [31]. It has previously been demonstrated that HepG2 cells principally express AMPKβ1, whereas HeLa cells express both AMPKβ1 and AMPKβ2 [36], yet the proportion of AMPKβ1/β2 complexes in endothelial cells has not been reported. The activities of AMPK complexes containing specific AMPKβ and α isoforms was therefore assessed in HAECs. Complexes containing AMPKβ1 accounted for approximately 60% of the total cellular AMPK activity and AMPKβ2 the remaining 40% in HAECs. As previously reported, complexes containing AMPKα1 represent the majority (~95%) of total cellular AMPK activity in HAECs [21] (Figure 8).

## 3. Discussion

This study demonstrates that A769662 inhibited the effects of insulin on Akt phosphorylation and NO synthesis in ECs, but had no substantive effect on Akt phosphorylation in MEFs, HeLa or HepG2 cells. This action of A769662 was not attenuated when AMPK activity was inhibited, and was not recapitulated by the alternative AMPK activators compound **991** and AICAR, suggesting this is an AMPK-independent action of A769662. In addition, A769662 inhibited insulin-stimulated Erk1 phosphorylation in ECs and MEFs, an effect that was still apparent in MEFs lacking AMPK activity. Taken together, these data suggest an EC-specific action of A769662 on early insulin signalling that is independent of AMPK.

Previous studies have demonstrated multiple beneficial effects of AMPK activation on insulin signalling, lipid and plasma glucose levels [17,23,37,38]. In ECs, AMPK activation has been reported to stimulate NO synthesis and angiogenesis while inhibiting pro-inflammatory signalling and reactive oxygen species synthesis [10]. Most of these studies have used AMPK activators that either indirectly activate AMPK through the inhibition of mitochondrial ATP synthesis, such as metformin, berberine and resveratrol or mimic AMP, such as AICAR [39]. As a consequence, these AMPK activators also have numerous other effects that are not mediated by AMPK. In contrast, A769662 and compound **991** are direct allosteric activators of AMPK that selectively or show a bias toward activating AMPK complexes containing the β1 regulatory subunit isoform [31]. Few studies have examined the endothelial effects of A769662, although it has been reported to inhibit antioxidant gene expression [40,41], viability and proliferation [13,32] and proinflammatory signalling [12,42] in human ECs. Furthermore, compound **991** has been also reported to inhibit proinflammatory signalling in ECs [12].

It is, therefore, surprising that A769662 inhibited insulin-stimulated NO synthesis in HUVECs and HAECs, as many studies report that AMPK activation stimulates NO synthesis [10]. Indeed, A769662 stimulates activating eNOS Ser1177 phosphorylation in hearts and AICAR and resveratrol have been reported to improve impaired insulin-mediated vascular responses in rodents [43,44,45]. Endothelial AMPK activation is not always associated with eNOS phosphorylation or NO synthesis, however [46,47], and the lack of insulin-stimulated NO synthesis in A769662-stimulated HAECs and HUVECs is likely to be a consequence of reduced insulin-stimulated Akt phosphorylation, since Akt is required for the activation of eNOS by insulin [48]. A769662 also decreased IGF1-stimulated Akt phosphorylation. Insulin and IGF1 both activate Akt [49], differing only in the initial step, as they bind to the insulin receptor (IR) and IGF1 receptor (IGF1R) respectively. In addition, IGF1R and IR share enough structural similarity to allow interchangeable binding of IGF1 and insulin [49,50]. Although IGF1 has a higher affinity for IGF1R, IGF1 may still bind IR and vice-versa. This may explain why A769662 seems to have a more modest effect on IGF1-stimulated Akt Ser473 phosphorylation, as IGF1 signalling may recruit distinct populations of IR substrate (IRS) proteins compared to insulin [51]. On the other hand, unlike insulin and IGF1, VEGF signalling does not utilise IRSs to recruit PI3K and was unaffected by A769662 preincubation [52]. As both Akt Ser473 and Thr308 phosphorylation were inhibited by A769662 in ECs, this suggests that A769662 does not simply inhibit the kinases that phosphorylate those sites, mammalian target of rapamycin complex 2 (mTORC2) or phosphoinositide-dependent protein kinase-1 (PDK1) respectively [2]. In addition, A769662 also inhibited insulin-stimulated Erk1/2 phosphorylation in ECs and MEFs, which is activated by a pathway separate to that of Akt after insulin receptor activation [53]. Importantly, A769662 also inhibited insulin-stimulated Erk1/2 phosphorylation in MEFs lacking AMPK, demonstrating that this effect is AMPK-independent. A769662 has previously been demonstrated to have no direct effect on the activity of Akt or PDK1, although it did inhibit the Erk1 kinase, mitogen-activated protein kinase kinase-1 (MKK1), in vitro [54]. In contrast, IGF-1-stimulated Erk1 phosphorylation was accentuated by A769662 in HUVECs, such that direct inhibition of MKK1 by A769662 may not simply underlie the inhibition of insulin-stimulated Erk1/2 phosphorylation. Taken together, these data indicate that A769662 inhibits at the level of the insulin/IGF-1 receptor or a receptor-associated protein, thereby inhibiting both pathways.

The inhibition of insulin-stimulated Akt phosphorylation by A769662 in ECs was not recapitulated by either compound 991 or AICAR, both of which activate AMPK to a similar degree. Compound **991** allosterically activates AMPK at a similar site to A769662 [31], whereas AICAR is phosphorylated to the AMP-mimetic, ZMP within cells [39]. Indeed, AICAR increased basal and insulin-stimulated Akt phosphorylation in ECs, in agreement with previous reports [15,21]. As both Akt Ser473 and Thr308 phosphorylation increased, this likely reflects stimulation of PI3K or earlier signalling events by AICAR, independent of AMPK activation. Given that the inhibition of Akt phosphorylation was still observed in ECs in which AMPK activity had been completely inhibited by SBI-0206965 and the lack of effect of compound **991** despite the similar mechanism by which compound **991** activates AMPK, these data indicate an AMPK-independent action of A769662 on insulin-stimulated Akt phosphorylation, although it occurs with a similar concentration to that required for AMPK activation.

Intriguingly, the inhibitory effect of A769662 on insulin-stimulated Akt activation was limited to ECs, as it was not observed in MEFs, HeLa or HepG2 cells, although the statistical significance of stimulation by insulin was lost in HeLa cells preincubated with A769662. A769662 (100 µM) has been demonstrated previously to inhibit basal Akt Ser473 phosphorylation in prostate cancer cell lines, yet stimulated Akt Ser473 phosphorylation at a lower concentration (50 µM) [55]. Indeed, previous studies have shown no effect of A769662 on insulin-stimulated Akt activation in rat adult cardiomyocytes, human myotubes or L6 cells [29,56,57]. In contrast to this neutral effect reported in striated muscle cells, A769662 has also been reported to stimulate glucose uptake in muscle cells by increasing PI3K association with IRS1, suggesting an AMPK-independent effect that increased Akt activity [58]. In addition, high concentrations of A769662 stimulated Akt phosphorylation in a manner sensitive to the PI3K inhibitor, wortmannin in CHO cells expressing the δ-opioid receptor [59]. It is clear, therefore, that A769662 influences Akt differentially in different cell types, providing further evidence that these effects are not mediated by AMPK.

In addition, we demonstrate that preincubation of HeLa cells, but not HepG2 cells or ECs, with compound **991** increased insulin-stimulated Akt phosphorylation. These data argue for a cell-type specific/selective effect of A769662 on insulin signalling, whereby only A769662 markedly reduces insulin signalling in ECs and insulin-stimulated Erk1/2 phosphorylation in ECs and MEFs. This cell-type selectivity could be related to differential expression of IR/IGF1R between cell types. HUVECs have been previously reported to have approximately 400,000 IGF1R/cell and 40,000 IR/cell [30]. Furthermore, according to the updated version of Human Protein Atlas (https://www.proteinatlas.org/) [60], HepG2 and HeLa cells have been reported to express 21.9 and 27.5 IGF1R transcripts per million (TPM), respectively and 15.9 and 1.9 IR TPM respectively [61]. Higher IR levels could preserve insulin-stimulated Akt and Erk1 phosphorylation by increasing the number of activated receptors and signal intensity. The high relative IR expression levels in HepG2 cells may therefore explain why A769662 had no effect on attenuation of insulin signalling in this cell line, whereas in HUVECs and HAECs, which have lower IR expression levels, there was such a marked effect of A769662.

It is unlikely that the differential effects of A769662 in the different cell types can be explained by differences in AMPK isoform expression, due to the selectivity of A769662 and compound **991** [31]. Both compounds would be expected to activate AMPK complexes containing β1, and complexes containing AMPKβ1 were found to contribute ~60% of the total cellular AMPK activity in HAECs. It has previously been reported that HepG2 cells principally express AMPKβ1, whereas HeLa cells express both AMPKβ1 and AMPKβ2 [36]. This further indicates that the inhibition of insulin signalling in ECs by A769662 is an AMPK-independent effect and unrelated to differential actions on specific pools of AMPK within ECs, as all the cell types investigated express abundant AMPKβ1 levels. As AMPK-independent effects of A769662, including inhibition of 26S proteasome activity in MEFs, voltage-gated Na^+^ channels in rat neurons and the Na^+^/K^+^ ATPase in L6 cells [26,28,29] have been reported previously, inhibition of insulin signalling in ECs may similarly be considered AMPK-independent. In addition, A769662 was recently reported to promote vasorelaxation in rabbit and rat arteries by reducing cytosolic Ca^2+^ levels, by an endothelium-dependent yet AMPK-independent manner [62].

In conclusion, A769662 decreases insulin-stimulated NO synthesis and Akt Ser473 phosphorylation in HUVECs and HAECs in a manner likely to be independent of AMPK. Furthermore, A769662 decreases insulin-stimulated Erk1/2 phosphorylation in a manner that is AMPK-independent in MEFs and HAECs. Taken together, these data demonstrate that caution should be exercised when interpreting data obtained using A769662 as a tool in cultured human endothelial cells.

## 4. Materials and Methods

### 4.1. Materials

Cryopreserved HUVECs, HAECs and MV2 medium were purchased from Promocell (Heidelberg, Germany). SV40-immortalised wild-type and AMPKα1 and AMPKα2 knock-out MEFs were kindly provided by Dr. B. Viollet (Institut Cochin, Paris, France) and have been described previously [33]. HeLa and HepG2 cells were obtained from ATCC (Manassas, VA, USA). IGF1, VEGF, SBI-0206965 and porcine insulin were purchased from Sigma Aldrich (St. Louis, MO, USA). AICAR was purchased from Toronto Research Chemicals Inc. (Ontario, ON, Canada). Compound **991** was synthetised by MRC Technology. A769662 and mouse anti-β-tubulin (#11307) antibodies were purchased from Abcam (Cambridge, UK). Rabbit anti-phospho-Akt Thr308 (#13038), anti-phospho-Akt Ser473 (#4058), anti-ERK1/2 (#9102), anti-phospho-ACC Ser79 (#3661), anti-ACC (#3676), anti-α-tubulin (#2144) and mouse anti-Akt (#2920) and anti-phospho-p44/42 Erk1/2 (Thr202/Tyr204) (#9106) antibodies were from New England Biolabs UK (Hitchin, UK). Sheep anti-AMPKα1, anti-AMPKα2, anti-AMPKβ1 and anti-AMPKβ2 antibodies used for immunoprecipitation of AMPK complexes containing specific subunit isoforms were a kind gift from Professor D.G. Hardie (University of Dundee) and have been described previously [39,63,64]. IRdye680 or 800-labelled donkey anti-mouse IgG (#926-32212) and anti-rabbit IgG (#926-68023 and #926-32213) antibodies were from LI-COR Biosciences (Lincoln, UK). Medium 199, RPMI 1640 and DMEM (4.5 g/L glucose) were from Life Technologies (Paisley, UK).

### 4.2. Cell Culture and Experimental Design

HAECs and HUVECs were cultured in MV2 medium supplemented with EC growth factor mix, 5% (*v*/*v*) serum (Promocell). HUVECs and HAECs were utilised between passages 3 and 6. MEFs and HeLa cells were cultured in DMEM supplemented with 10% (*v*/*v*) foetal calf serum. HepG2 cells were cultured in RPMI 1640 supplemented with 10% (*v*/*v*) foetal calf serum, 1 mM sodium pyruvate. Once cells reached 90–100% confluence, HUVECs/HAECs were incubated in serum-free Medium 199 and HeLa cells and HepG2 cells were incubated with serum-free DMEM for 2 h and then with AMPK activators prior to stimulation with insulin (1 μM), VEGF (10 ng/mL) or IGF1 (25 ng/mL). Cells were placed on ice and washed with PBS prior to lysis in Triton X-100-based lysis buffer (50 mM Tris-HCl, pH 7.4 at 4 °C, 50 mM NaF, 1 mM Na_4_P_2_O_7_, 1 mM EDTA, 1 mM EGTA, 1% (*v*/*v*) Triton-X-100, 250 mM mannitol, 1 mM DTT, 1 mM Na_3_VO_4_, 0.1 mM benzamidine, 0.1 mM PMSF, 5 μg/mL SBTI). Cell lysates were scraped into microcentrifuge tubes and incubated on ice for 20 min, centrifuged (5 min, 21,910× *g*, 4 °C) and the subsequent supernatants stored at −20 °C. Lysate protein concentrations were determined using Bradford or BCA methods, as previously described [6,21].

### 4.3. SDS-Polyacrylamide Gel Electrophoresis and Immunoblotting

Cell lysate proteins were resolved by SDS-PAGE and immunoblotted with antibodies diluted in 50% (*v*/*v*) LI-COR blocking buffer in TBS containing 0.1% (*v*/*v*) Tween-20 as described previously [13]. Proteins were visualised using infrared dye-labelled secondary antibodies on a LI-COR Odyssey infrared imaging system and analysed using the ImageJ software for densitometric quantification of band intensity. In some cases, indicated in the figure legends, immunoblots were stripped in 0.2 M NaOH for 10 min, washed multiple times with Tris-buffered saline (TBS) until the pH returned to 7.2, and then blocked in TBS supplemented with 5% (*w*/*v*) milk powder prior to probing with primary antibodies. In all other cases, immunoblots for phospho- and total protein levels of ACC, Akt and Erk1/2 were obtained on immunoblots conducted concurrently in parallel.

### 4.4. NO Assay

HAECs and HUVECs were cultured in 6-well plates until 100% confluent and serum-starved for 2 h in Medium 199 and then in the presence of Krebs-Ringer-Hepes (KRH) buffer for 20 min. The medium was replaced and cells incubated in the presence or absence of stimuli in KRH for a further 15–20 min. Samples of medium (50 μL) were taken at different intervals and 200 μL methanol added to each. Samples were centrifuged (21,910× *g*, 4 °C, 20 min), and supernatants were stored at −20 °C. NO concentration was determined using a Sievers 280A NO Meter (Sievers, Boulder, CO, USA) as described previously [21].

### 4.5. AMPK Assay

AMPK specific isoforms were immunoprecipitated from HAECs using sheep anti-AMPKα1, anti-AMPKα2 [63], anti-AMPKβ1 [39] or anti-AMPKβ2 [64] antibodies bound to protein G-Sepharose (1 µg antibody and 5 µL packed volume protein G-Sepharose/immunoprecipitation) in IP buffer (50 mM Tris-HCl (pH 7.4 at 4 °C), 150 mM NaCl, 50 mM NaF, 5 mM Na_4_P_2_O_7_, 1 mM EDTA, 1 mM EGTA, 1% (*v*/*v*) Triton-X-100, 1% (*v*/*v*) glycerol, 1 mM DTT, 0.1 mM benzamidine, 1 mM PMSF, 5 μg/mL SBTI, 1 mM Na_3_VO_4_). Immunoprecipitates were then washed into HBD buffer (50 mM HEPES-NaOH (pH 7.4), 0.02% (*v*/*v*) Brij-35, 1 mM DTT) and assayed for AMPK activity using SAMS substrate peptide as described previously [12,35].

### 4.6. Statistical Analysis

All data is expressed as a relative change (mean ± SEM) from control baseline or normalised to group of reference for each experiment. Prism software (GraphPad Software, San Diego, CA, USA) was used to perform one or two-way ANOVA (with Tukey’s or Dunnett’s post hoc multiple comparison tests), where appropriate, using *p* < 0.05 as significant.

## Supplementary Materials

The following are available online at http://www.mdpi.com/1422-0067/19/12/3886/s1.
