# Supplementary material for: A769662 Inhibits Insulin-Stimulated Akt Activation in Human Macrovascular Endothelial Cells Independent of AMP-Activated Protein Kinase
Source: Int J Mol Sci. 2018 Dec 5;19(12):3886. doi: 10.3390/ijms19123886 (PMC6321332; doi:10.3390/ijms19123886)
Supplement: Supplementary file 1 [file ijms-19-03886-s001.pdf]

Supplementary: A769662 Inhibits Insulin-Stimulated Akt Activation in Human Macrovascular Endothelial Cells Independent of AMP-Activated Protein Kinase

Anastasiya Strembitska [1], Sarah J. Mancini [1], Jonathan M. Gamwell [1,2], Timothy M. Palmer [1,3], George S. Baillie [1] and Ian P. Salt [1,*]

1 Institute of Cardiovascular and Medical Sciences, College of Medical, Veterinary and Life Sciences, University of Glasgow, Glasgow G12 8QQ, UK; a.strembitska.1@research.gla.ac.uk (A.S.); sarah.mancini@glasgow.ac.uk (S.J.M.); jonathan.gamwell@rdm.ox.ac.uk (J.G.); tim.palmer@hyms.ac.uk (T.M.P.); george.baillie@glasgow.ac.uk (G.S.B.)
2 Radcliffe Department of Medicine, University of Oxford, Oxford OX3 9DU, UK
3 Centre for Atherothrombosis and Metabolic Disease, Hull York Medical School, University of Hull, Hull HU6 7RX, UK
* Correspondence: ian.salt@glasgow.ac.uk; Tel.: +44-(0)141-330-2049; Fax: +44-(0)141-330-5481

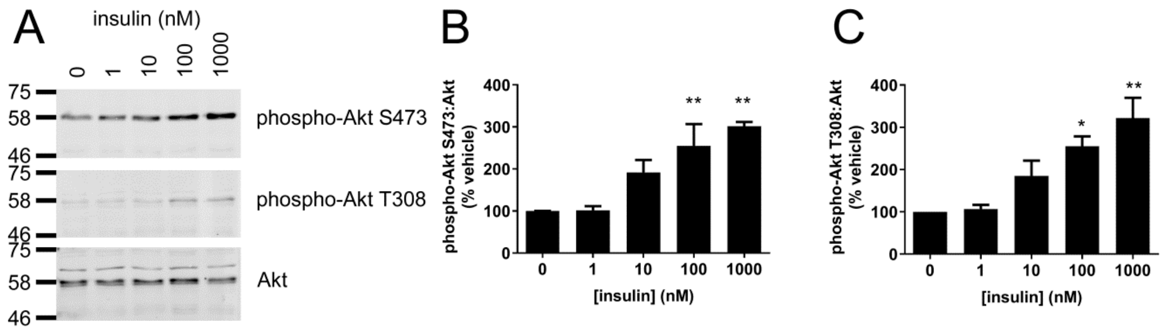

Figure S1: Concentration-dependence of insulin-stimulated Akt phosphorylation in HUVECs. HUVECs were stimulated with the indicated concentrations of insulin for 15 min, cell lysates were prepared, proteins resolved by SDS-PAGE and subjected to immunoblotting with the antibodies indicated. (A) Representative immunoblots are shown from three independent biological replicates with molecular weight markers in kDa indicated. (B, C) Densitometric quantification (mean ± SEM) of immunoblots of (B) Akt Ser473 and (C) Akt Thr308 phosphorylation normalised to total Akt levels. *p<0.05, **p<0.01 relative to absence of insulin.

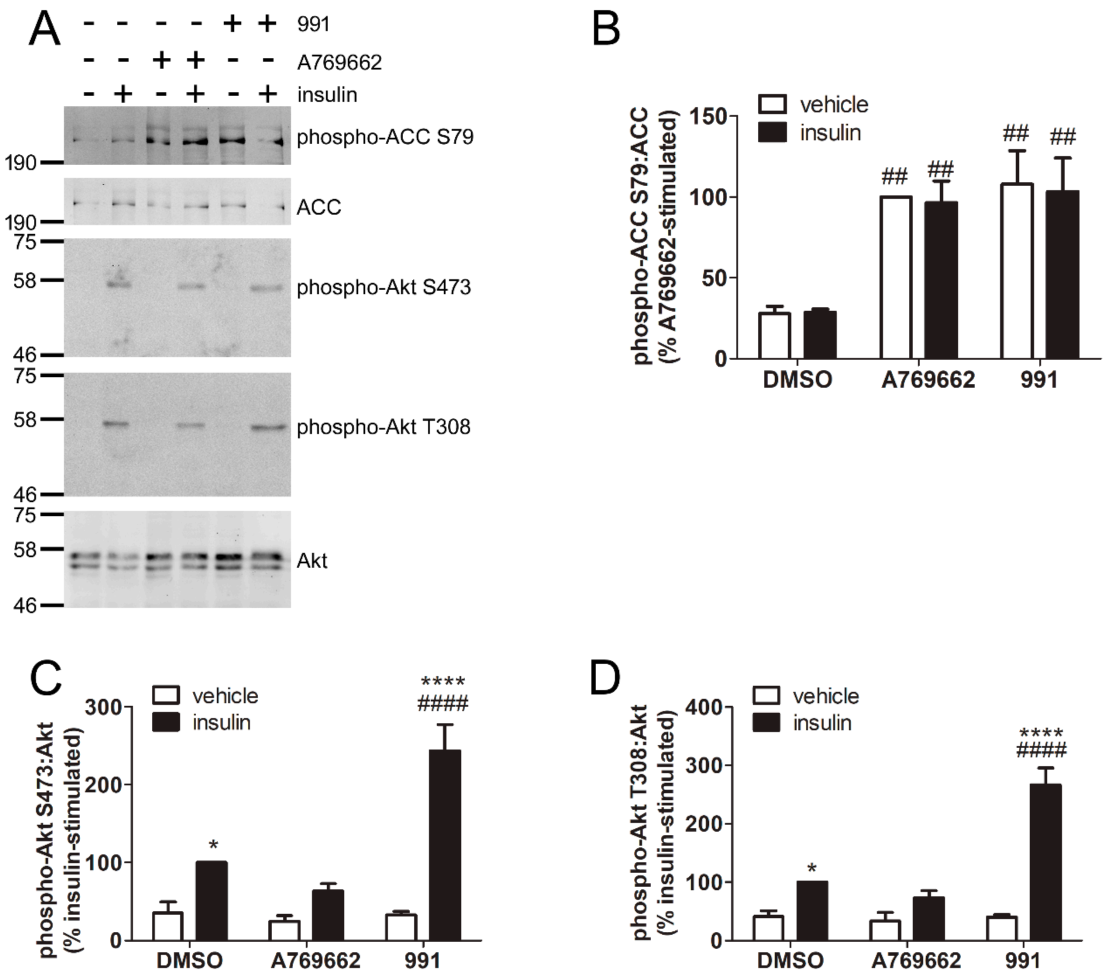

Figure S2**: The effect of A769662 on insulin-stimulated on Akt phosphorylation in HeLa cells.** HeLa cells were stimulated with A769662 (50 μM, 45 min) or compound 991 (5 μM, 60 min) prior to insulin (1 μM, 15 min). Cell lysates were prepared, proteins resolved by SDS-PAGE and immunoblotted with the antibodies indicated. (A) Representative immunoblots from three independent biological replicates with molecular weight markers indicated. Total ACC and phospho-Akt Thr308 protein levels were assessed by stripping and re-probing the membranes. (B-D) Densitometric quantification of (B) ACC Ser79, (C) Akt Ser473 or (D) Akt Thr308 phosphorylation normalised to total ACC or Akt levels respectively (mean ± SEM). *$p<0.05$, ****$p<0.0001$, relative to absence of insulin. ##$p<0.01$, ####$p<0.0001$ relative to absence of AMPK activator.

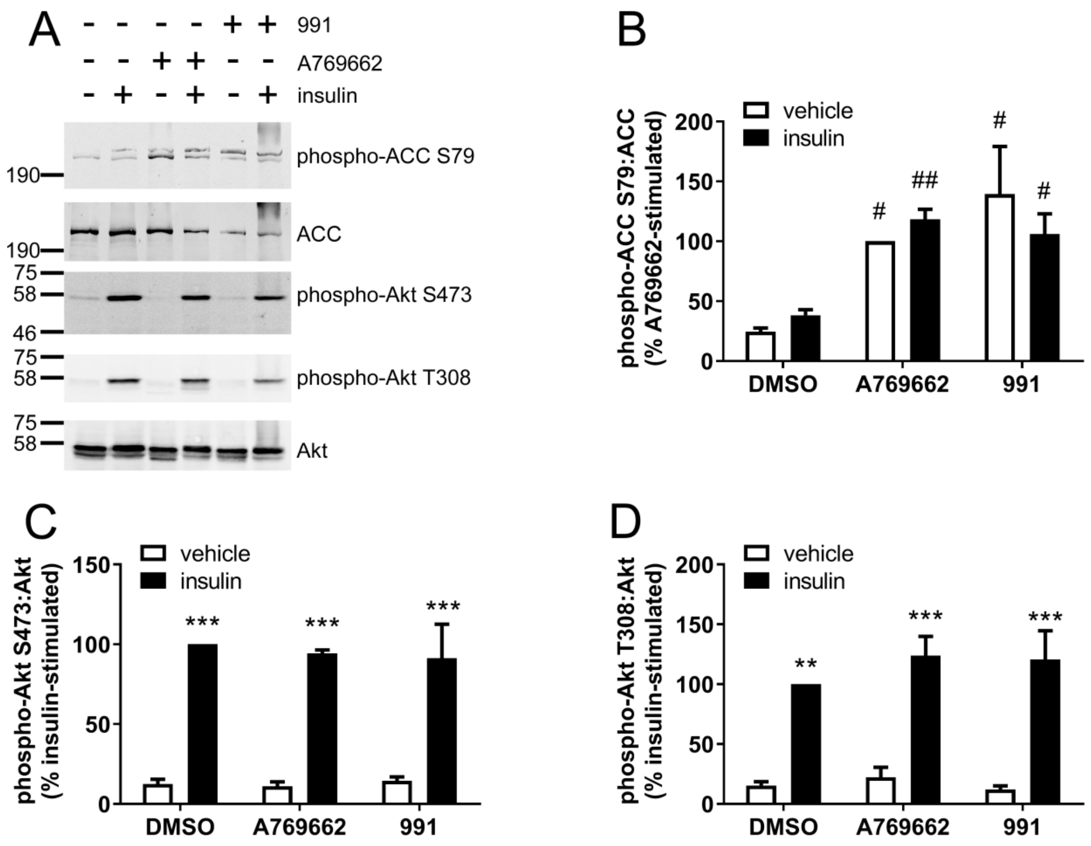

Figure S3: The effect of A769662 on insulin-stimulated on Akt Ser473 phosphorylation in HepG2 cells. HepG2 cells were stimulated with A769662 (50 μM, 45 min) or compound 991 (5 μM, 60 min) prior to insulin stimulation (1 μM, 15 min). Cell lysates were prepared, proteins resolved by SDS-PAGE and immunoblotted with the antibodies indicated. (A) Representative immunoblots from three independent biological replicates with molecular weight markers indicated. Total ACC and phospho-Akt Thr308 protein levels were assessed by stripping and re-probing the membranes. (B-D) Densitometric quantification (mean ± SEM) of (B) ACC Ser79, (C) Akt Ser473 or (D) Akt Thr308 phosphorylation normalised to total ACC or Akt levels respectively (mean ± SEM). **$p<0.01$, ***$p<0.001$ relative to absence of insulin. #$p<0.05$, ##$p<0.01$ relative to absence of AMPK activator.
